# Thermocompressed Chickpea-Flour Sheets Reinforced with Cellulose Nanocrystals: Improved Water-Vapor Barrier and Thermo-Mechanical Performance

**DOI:** 10.3390/polym18101175

**Published:** 2026-05-10

**Authors:** Emmanuel Flores-Huicochea, Magarito Somera-González, Monserrat Morales-Catalán, Claudia Andréa Romero-Bastida, Allison Vianey Valle-Bravo, Carlos López-González, Amalia Irais Cuno-Jaimes, Rosalía América González-Soto

**Affiliations:** CeProBi, Instituto Politécnico Nacional, Carr. Yautepec-Jojutla km. 6.5, Col. San Isidro, Yautepec 62739, Morelos, Mexico; efloresh@ipn.mx (E.F.-H.); msomera2200@alumno.ipn.mx (M.S.-G.); mmoralesc2102@alumno.ipn.mx (M.M.-C.); cbastida@ipn.mx (C.A.R.-B.); avalleb2103@alumno.ipn.mx (A.V.V.-B.); clopezgo@ipn.mx (C.L.-G.); acuno@ipn.mx (A.I.C.-J.)

**Keywords:** thermocompression, chickpea flour, cellulose nanocrystals, nanocomposite sheets, water-vapor permeability, X-ray diffraction, thermal stability

## Abstract

Chickpea (*Cicer arietinum* L.) flour is a promising raw material for bio-based packaging due to its protein and polyphenol content. In this study, thermocompressed chickpea flour sheets were reinforced with cellulose nanocrystals (CNCs) to improve their barrier, mechanical, optical, thermal, and structural properties. Preliminary trials identified 22% moisture as the most suitable condition for consistent sheet formation. CNC was incorporated at 0, 2.5, 5.0, and 7.5% (*w*/*w*). Thermocompression reduced the measurable phenolic fractions, although antioxidant activity was not significantly affected. CNC markedly reduced water vapor permeability from 5.16 × 10^−10^ in the control to 5.93 × 10^−12^ g∙m^−1^∙s^−1^∙Pa^−1^ at 7.5% CNC. Tensile strength and Young’s modulus increased with CNC loading, whereas elongation at break was highest at intermediate concentrations. Optical characterization showed changes in transmittance and opacity. Thermal analysis indicated that CNC modified the DSC thermal event, whereas only minor differences were observed in the TGA profile. SEM, DSC, XRD, and FTIR analyses suggested changes in morphology and thermo-structural organization. Overall, CNC improved barrier and mechanical performance, supporting the potential of these sheets as a material for semirigid biodegradable packaging applications.

## 1. Introduction

Food packaging is a key technological component for preserving quality and safety by limiting moisture and gas transfer, protecting foods against mechanical damage, and reducing microbial contamination [1,2]. Conventional petroleum-derived plastics remain dominant due to their performance and low cost; however, their persistence in the environment and end-of-life challenges have accelerated research into bio-based polymer systems and sustainable polymer processing routes [3]. The shift from conventional petroleum-derived plastics to bio-based, biodegradable polymers in food packaging is driven by the need to address environmental concerns and reduce plastic waste [4]. In this context, bio-based and biodegradable packaging materials have attracted attention as alternatives to reduce dependence on fossil fuels and mitigate plastic waste [4].

Biopolymer matrices derived from proteins and polysaccharides can form coherent networks. However, their performance is often constrained by moisture sensitivity and structure–property trade-offs [5,6]. Protein-based systems typically provide good oxygen barrier performance but may exhibit plasticization and loss of stiffness at higher water activities [7]. Polysaccharide-rich matrices can exhibit favorable gas barrier properties. However, water-vapor barrier performance is often inadequate for practical packaging conditions [5]. Flour-based formulations are attractive polymeric feedstocks because they integrate proteins, carbohydrates, and minor lipids into a single ingredient and can carry phenolic compounds that may provide added functionality [6]. Among legume flours, chickpea (*Cicer arietinum* L.) is notable for its high protein content and compositional complexity, which can promote network formation under thermal and mechanical treatment [7,8].

From a polymer-processing perspective, most bio-based packaging materials reported at laboratory scale are prepared by casting [9]. In contrast, industrial manufacturing relies on thermal–mechanical routes (e.g., extrusion and compression-based forming) that enable higher throughput and improved thickness control [10,11]. Thermocompression is a relevant alternative because it consolidates the material under heat and pressure, potentially enhancing interfacial contact, densification, and the development of physical interactions within the protein–polysaccharide network [10,12]. Nevertheless, moisture transport remains a central limitation in flour-based matrices, and strategies that rationally modify microstructure and chain mobility are required to achieve better barrier and thermo-mechanical performance [13,14].

CNC is a promising nanofiller for bio-based polymer composites due to its high crystallinity, large specific surface area, and hydroxyl-rich surfaces that can interact with hydrophilic matrices [15,16]. When appropriately incorporated, CNC can increase network cohesion and promote tortuous diffusion pathways, thereby reducing water-vapor permeability and potentially improving thermal stability through restricted segmental mobility and increased structural ordering [16]. Significantly, the efficacy of CNC reinforcement depends on processing history, filler loading, and dispersion state, which jointly control microstructure and macroscopic properties [16,17,18].

In this work, we use the term “sheets” rather than “films” because thermocompression typically produces structures with thicknesses above 0.25 mm, whereas casting commonly produces films below this threshold [19,20]. However, the performance of the thermocompressed chickpea-flour sheet reinforced with CNC remains under-reported, particularly regarding barrier and thermo-structural responses under controlled formulation and processing conditions. We hypothesized that CNC would create more tortuous diffusion pathways and a more ordered network, thereby improving WVP and thermal stability. Therefore, this work evaluates the effect of CNC loading on WVP, optical properties, thermal behavior, X-ray diffraction response, and morphology of the thermocompressed chickpea-flour sheets, while also verifying whether thermocompression conditions preserve phenolic content and antioxidant activity.

Although CNCs have been widely studied as reinforcing agents in bio-based materials, limited information is available on their effects in thermocompressed chickpea flour sheets, particularly regarding the combined modification of mechanical, barrier, thermal, and structural properties. In addition, most previous studies have focused on cast films or other polymeric matrices, while the behavior of chickpea flour systems processed by thermocompression remains less explored. Therefore, this study aimed to evaluate the influence of varying CNC concentrations on the physicochemical, mechanical, optical, thermal, and structural properties of thermocompressed chickpea flour sheets, in order to assess their potential as a biodegradable material for semirigid packaging applications, particularly where improved water-vapor barrier performance and moderate rigidity may be advantageous.

## 2. Materials and Methods

### 2.1. Materials

Chickpea flour (*Cicer arietinum* L.) was obtained from Harinas Viesca (CDMX, Mexico). Glycerol, sulfuric acid, and methanol were purchased from Fermont (Monterrey, Mexico). Microcrystalline cellulose (Catalog Number 435236) and 2,2-diphenyl-1-picrylhydrazyl (DPPH, Catalog Number D-9132) were purchased from Sigma-Aldrich Chemistry (St. Louis, MO, USA).

### 2.2. Experimental Design and Formulations

Preliminary tests were conducted at four moisture levels (18, 20, 22, and 24%) to establish workable dough moisture contents and sheet-forming conditions. Moisture contents of 18% and 24% (*w*/*w*) were discarded because the dough was either excessively dry or overly humid. Therefore, only 20% and 22% (*w*/*w*) were selected for pilot sheet formation by thermocompression. Based on handling and lamination performance during thermocompression, 22% moisture was selected as the reference condition for subsequent CNC-reinforced formulations.

To study reinforcement effects, CNC was incorporated at 2.5, 5.0, and 7.5% (*w*/*w*). The non-reinforced chickpea sheet was used as a control (CS), and reinforced sheets were labeled CSN.

### 2.3. Preparation of Cellulose Nanocrystals

Cellulose nanocrystals (CNCs) were prepared from microcrystalline cellulose by sulfuric acid hydrolysis following the method reported by Lapuz et al. [21] with modifications. Briefly, microcrystalline cellulose was hydrolyzed with 64% sulfuric acid at 45 °C for 45 min, using a cellulose-to-acid ratio of 10 mg cellulose per g acid solution. The suspension was diluted with cold deionized water (1:10, *v*/*v*) at 4 °C. The suspension was then centrifuged at 3200 rpm to remove the excess acid and recover the solid fraction.

The resulting suspension was dialyzed against deionized water using regenerated cellulose membranes (MWCO 12 kDa) until a neutral pH was reached, with water changes every 8 h. The dialyzed suspension was subsequently sonicated using a Hielscher UP200St ultrasonic processor (Teltow, Germany) equipped with a BS4d34 sonotrode (34 mm diameter) for 30 min at 40% amplitude, in continuous mode, using a 100 mL processing volume and an immersion depth of 25 mm. Sonication was performed in an ice bath to minimize temperature increases during treatment.

The sonicated suspension was filtered through a Gooch crucible with a 4.0–5.5 µm pore size, and the recovered material was dried at 60 °C for 4 h. The dried CNCs were stored for subsequent characterization and incorporation into the sheet formulations.

### 2.4. Transmission Electron Microscopy (TEM) and Nanoparticle Size Distribution Analysis

The morphology of CNC was evaluated by TEM (JEOL JEM-2100, Germany). Prior to observation, the CNC suspension was diluted with deionized water and was sonicated for 30 min (ultrasonic cleaner, Vevor, Shanghai, China). A drop of diluted CNC suspension was deposited onto a carbon-coated copper grid for TEM analysis. The sample was allowed to dry before imaging.

Nanoparticle dimensions were measured from the TEM micrographs using Digital Micrograph software (Gatan, USA v. 3.x). A total of 70 nanoparticles were analyzed in terms of length, width, and width-to-length ratio. For each variable, descriptive statistics were computed, and the data distribution was evaluated using histograms with 14 class intervals. To provide a smoothed representation of the distribution, kernel density curves were superimposed on the histograms. The histograms were expressed on a density scale, so that the height of each bar was defined as:

(1) $$d_{i}=\frac{n_{i}}{Nh_{i}}$$

where $d_{i}$ is the density of class i, $n_{i}$ is the number of observations in that class, N is the total number of observations, and $h_{i}$ is the class interval width. Under this normalization, the total histogram area is equal to 1. Additionally, the kernel density estimates were expressed as:

(2) $$f(x)=\frac{1}{Nh}\sum_{j=1}^{N}K\left(\frac{x-x_{j}}{h}\right)$$

where $f\left(x\right)$ is the estimated density, K is the kernel function, h is the smoothing parameter, and $x_{j}$ represents each observation. This analysis enabled visualization of relative concentration, dispersion, and possible asymmetry of nanoparticle dimensions. All graphs and descriptive statistical analyses were performed in R [22] using the ggplot2 package [23].

### 2.5. Preparation of Chickpea Flour Sheets by Thermocompression

Sheets were prepared from 100 g of chickpea flour, which was blended with glycerol (25% *w*/*w*) and distilled water (20 or 22% *w*/*w*). The mixture was homogenized using a mixer and stored at 4 °C for 24 h to improve component equilibration and dough homogeneity. The resulting dough was laminated with a roller to reduce thickness and cut into 10 cm × 10 cm sheets.

The thermocompression was carried out using a Carver hydraulic press (Model 3851-0, Wabash, IN, USA). Press platens were preheated to 135 °C. Each sheet was first placed between plates for 2 min without pressure, and then the pressure was increased to 8500 psi for 2 min. Pressure was released for 20 s, and the pressurization cycle was repeated twice. Finally, pressure was released, the press was turned off, and sheets were allowed to cool for 15 min inside the equipment before being removed and stored for subsequent analyses.

CNC-reinforced sheets were prepared following the same procedure, with the incorporation of 2.5, 5.0, or 7.5% (*w*/*w*) CNC.

### 2.6. Phenolic Compounds and Antioxidant Capacity

#### 2.6.1. Extraction of Phenolic Compounds

Free phenolic compounds were extracted according to the method described by Pérez-Jiménez & Saura-Calixto [24]. For polar free phenolics, 0.5 g of the sample (flour or sheet) was mixed with 10 mL of methanol/water (50:50, *v*/*v*), shaken in the dark for 1 h, and centrifuged at 3000 rpm for 10 min; the supernatant was collected. Subsequently, the residues were extracted to recover non-polar free phenolics using 10 mL of acetone/water (70:30, *v*/*v*) under the same conditions. Both supernatants were combined and brought to a final volume of 25 mL.

#### 2.6.2. Extraction of Bound Phenolics (Condensed and Hydrolyzable Tannins)

For the extraction of condensed tannins, 200 mg of the residue after the polar extraction was mixed with 10 mL of butanol/HCl/FeCl_3_ solution, agitated for 3 h, and centrifuged at 3000 rpm for 10 min. The supernatant was collected, and the residue was then washed with an additional 10 mL of the same solution. Both supernatants were then combined [25].

For the extraction of hydrolyzable tannins, 200 mg of the polar-extraction residue was mixed with 20 mL of methanol and 2 mL of concentrated sulfuric acid, and the mixture was incubated at 85 °C for 20 h. After incubation, the sample was centrifuged at 3000 rpm for 10 min, and the supernatant was collected. The residue was washed with 10 mL of water, centrifuged, and the resulting supernatant was combined with the first extract to obtain a final volume of 50 mL [26].

#### 2.6.3. Determination of Phenolic Compounds (Folin–Ciocalteu)

The total phenolic compounds were determined using the Folin–Ciocalteu assay, following the method described by Singleton et al. [27] and Singleton and Rosi [28]. Succinctly, 0.5 mL of the extract was mixed with 0.5 mL of Folin–Ciocalteu reagent and allowed to stand for 5 min. Subsequently, 10 mL of sodium carbonate (7.5%) and 14 mL of distilled water were added. After incubation in the dark for 2 h, the absorbance was measured at 765 nm. Free phenols were expressed as mg gallic acid equivalents (GAE)/g sample.

Condensed tannins were quantified using the butanol-HCl-Fe assay [25], and absorbance was measured at 555 nm. The results were expressed as mg catechin equivalents (CAE)/g sample.

Hydrolyzable tannins obtained after methanol/sulfuric acid hydrolysis were quantified at 750 nm using the Folin-based assay [26] and expressed as mg gallic acid equivalents (GAE)/g sample.

#### 2.6.4. Antioxidant Capacity (DPPH Scavenging)

The DPPH (2,2-diphenyl-1-picrylhydrazyl) assay is based on the reduction of the stable purple DPPH• radical by antioxidant compounds, resulting in a measurable decrease in absorbance at 517 nm. Radical scavenging activity (RSA) was determined using DPPH according to the method of Byun, Kim, and Whiteside [29,30,31].

Briefly, approximately 100 mg of sheet was placed in 2 mL of methanol and stirred for 3 h at 20 °C. The resulting supernatant (500 µL) was analyzed by mixing 2 mL of DPPH methanolic solution (0.06 mM). A control solution was prepared by replacing the sample extract with 500 µL of methanol. The mixtures were vortexed and incubated in the dark at room temperature for 30 min, after which the absorbance was recorded at 517 nm. The RSA was calculated using Equation (3).

(3) $$\%RSA=100\;\left(1-\frac{A_{S}}{A_{C}}\right)$$

where: $A_{S}$ is the absorbance of the sample, and $A_{C}$ is the absorbance of the control.

### 2.7. Thickness and Conditioning Prior to Testing

Sheet thickness was measured using a digital micrometer (Mitutoyo, model 293-230, Hiroshima, Japan) with a precision of 0.001 mm. Before mechanical and WVP tests, the sheets were conditioned for 2 days at 57% relative humidity in a chamber containing a saturated sodium bromide (NaBr) solution.

### 2.8. Mechanical Properties Characterization

Mechanical properties were determined using a TAX2i Texture Analyzer (Stable Micro Systems, Godalming, UK) equipped with a 25 kg load cell, following the corresponding ASTM standard [32]. Conditioned sheets were cut into 1 × 10 cm strips. Sheet thickness was measured at eight points using a micrometer, and the average value was used for calculations. The initial grip separation was 6 cm, and the test speed was 2.2 mm/s. Tensile strength, percent elongation at break (%), and Young’s modulus were determined using at least 10 replicates per formulation.

### 2.9. Water Vapor Permeability

WVP was determined gravimetrically according to ASTM E96/E96M-22ae1 [33] using stainless steel permeability cups. Conditioned sheets were cut into circular specimens (7.5 cm in diameter), and the thickness was measured at six points with a digital micrometer; the average thickness was used for the calculations. Each specimen was sealed over the cup opening, providing an exposed permeation area $\left(A\right)$ of 44.18 cm^2^.

The cups were filled to half capacity with silica gel to maintain 0% relative humidity (RH) inside the cell, and then placed in a desiccator containing saturated NaCl solution to maintain 75% RH outside the cup at 25 °C. Cup mass was recorded every hour for 8 h, and the linear region of the mass gain versus time plot was used to calculate the water vapor transmission rate (WVTR), according to Equation (4):

(4) $$WVTR=\frac{s}{A}$$

where s is the slope of the linear regression of mass gain versus time $\left(g\cdot s^{-1}\right)$, and A is the exposed area $\left(m^{2}\right)$. WVP was calculated as:

(5) $$WVP=\frac{WVTR\cdot L}{\Delta P}$$

where L is the average sheet thickness $\left(m\right)$, and ΔP is the water vapor partial pressure difference across the film $\left(Pa\right)$, calculated from the RH gradient between both sides of the specimen at the test temperature. Measurements were performed in triplicate.

### 2.10. Color Measurement

Color measurements were performed using a portable colorimeter (Konica Minolta CR-400, Tokyo, Japan), previously calibrated with a standard white plate (L = 97.37, a = −0.30, b = 2.49). Results were expressed in CIELAB coordinates (L*, a*, b*). For each sample, four measurements were taken at different locations on the sheet surface, and the average values were used for analysis. Chroma and hue angle were calculated from the a* and b* coordinates.

### 2.11. Transparency, Opacity

Transparency and opacity were determined from UV-Vis transmittance spectra according to the method reported by Aguirre et al. [34]. Sheets were cut to fit the sample holder, and measurements were taken at four different positions per formulation.

Light transmittance at 600 nm (%T600) was used as a direct descriptor of visible-light transmission through the sheets. Because thickness-normalized transparency expressions based on log(%T_600_)/x may lead to an ambiguous interpretation [35]. The optical behavior of the sheets was assessed using %T_600_ together with opacity.

The opacity was calculated as follows (Equation (6)):

(6) $$Opacity=\frac{A_{500}}{x}$$

where A_600_ is the absorbance at 600 nm, and x is the sheet thickness (mm). This parameter was used to compare the light attenuation behavior of the formulations on a thickness-normalized basis.

### 2.12. Thermal Properties, TGA and DSC

Thermogravimetric analysis (TGA) was performed using a TGA/DSC 2 STAR System (Mettler Toledo, Columbus, OH, USA). Samples were heated from 25 °C to 600 °C at 20 °C/min under nitrogen with a flow rate of 60 mL/min. The thermal parameters obtained from the TGA curves included the temperatures at 5% (T5%) and 10% (T10%) weight loss, the onset degradation temperature (Tonset), the temperature of maximum degradation rate (Tmax), and the residual mass at the final temperature. Tmax was determined from the derivative thermogravimetric (DTG) curve.

Differential Scanning Calorimetry (DSC) was performed using a TA Q-200 calorimeter (TA Instruments, New Castle, DE, USA) to identify the thermal transitions of the sheets. Samples were heated from 25 to 200 °C at 10 °C/min under a nitrogen atmosphere.

### 2.13. X-Ray Diffraction (XRD)

XRD patterns were collected using a Siemens D500 powder diffractometer (Fort Worth, TX, USA) operated at 35 kV and 25 mA with Cu Kα radiation in Bragg–Brentano geometry. The sheets were cut into suitable pieces and mounted flat on the sample holder to ensure a uniform surface during analysis. Diffraction patterns were recorded over a 2θ range of 5–40°, using a step size of 0.02° and a counting time of 1 s per step.

### 2.14. Scanning Electron Microscopy (SEM)

The sheet morphology was analyzed by SEM using a JEOL JSM-5410LV microscope (Tokyo, Japan). Samples were cut, mounted on 1 cm diameter cylindrical copper stubs, gold-coated, and observed under high-vacuum conditions.

### 2.15. FTIR Spectroscopy

FTIR spectra of the sample were obtained using an attenuated total reflectance (ATR) accessory over the spectral range of 400–4000 cm^−1^, with a resolution of 4 cm^−1^ and 64 scans per sample.

### 2.16. Statistical Analysis

Statistical analyses were performed in R (versión 4.5.0, R Foundation for Statistical Computing, Vienna, Austria) [22]. For sheet properties, results are presented as mean ± standard deviation. Differences among formulations with different CNC loadings (0, 2.5, 5.0, and 7.5% *w*/*w*) were evaluated using one-way analysis of variance (ANOVA), followed by Tukey’s honest significant difference (HSD) test when the main difference was significant. Letter-based groupings in tables and figures indicate statistically significant differences among means. The assumptions of normality and homogeneity of variances were assessed using Shapiro–Wilk and Levene’s tests, respectively. When these assumptions were not met, data were transformed prior to analysis. A *p*-value of < 0.05 was considered statistically significant.

For CNC morphometric data obtained from TEM images, length and width distributions were positively skewed; therefore, these variables were primarily summarized using the median (Q1–Q3), whereas mean ± standard deviation was included only as complementary information, with previous studies.

## 3. Results

Preliminary trials indicated that 22% moisture provided better handling and lamination during processing, resulting in more consistent sheet formation. Accordingly, this condition was selected for the subsequent experiment, and the following sections describe the effect of CNC loading on the structural and functional properties of the thermocompressed chickpea sheets.

### 3.1. Characterization of Cellulose Nanocrystals (CNCs)

**Figure 1:**
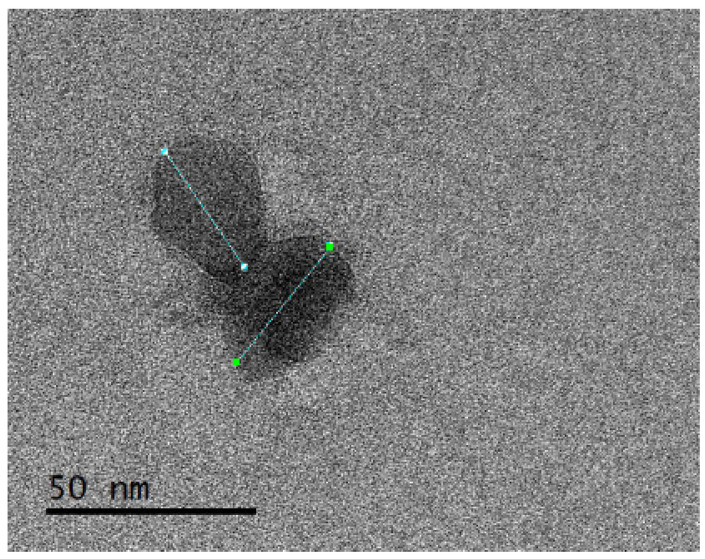
TEM micrography CNC (JEOL JEM-2100; 120,000×) obtained using JEOL JEM-2100 microscope at 120,000× magnification. Scale bar: 50 nm. Green markers indicate representative particle dimensions measured on the micrograph.

Transmission electron microscopy (TEM) confirmed that the obtained CNCs were in the nanometric range and exhibited individual particles with quasi-isometric to slightly elongated projections. However, some degree of particle overlap or aggregation was also observed (Figure 1). Morphometric analysis of TEM images showed that CNC dimensions were dispersed and right-skewed. Therefore, the median (Q1–Q3) was used as the primary descriptor for absolute particle dimensions. The median length was 8.94 nm (Q1–Q3: 6.20–29.94 nm), and the median width was 6.82 nm (Q1–Q3: 4.92–25.53 nm). The mean width-to-length ratio was 0.782 ± 0.040, indicating that particle width represented, on average, approximately 78% of particle length (Table 1).

The descriptive statistics revealed a wide range of absolute particle dimensions. Length ranged from 3.82 to 66.83 nm, while width varied from 2.86 to 58.21 nm, with high coefficients of variation (91.24% and 93.62%, respectively), indicating marked heterogeneity in particle size. In contrast, the width-to-length ratio showed a much narrower range (0.686–0.897) and substantially lower coefficient of variation (5.07%), indicating that particle proportions were more homogeneous than the absolute dimensions.

**Figure 2:**
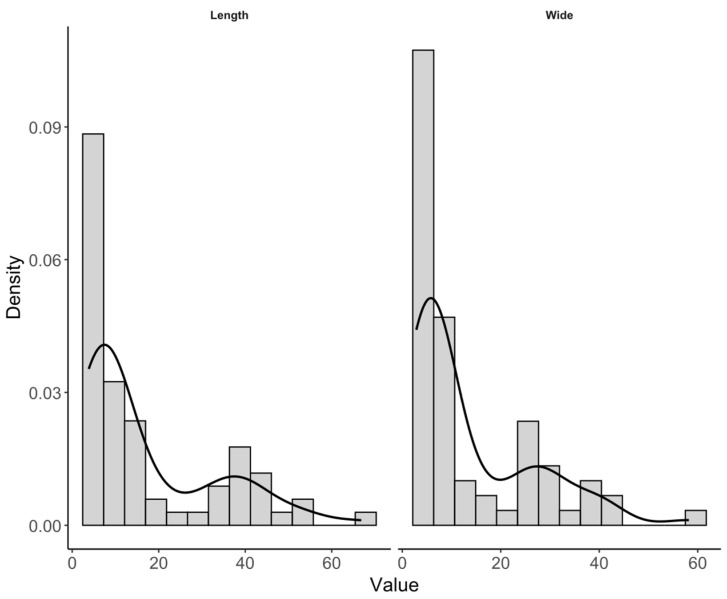
Distribution of cellulose nanocrystal (CNC) dimensions measured from TEM micrographs. Histograms and superimposed kernel density curves are shown for particle length and width, expressed on a density scale.

**Figure 3:**
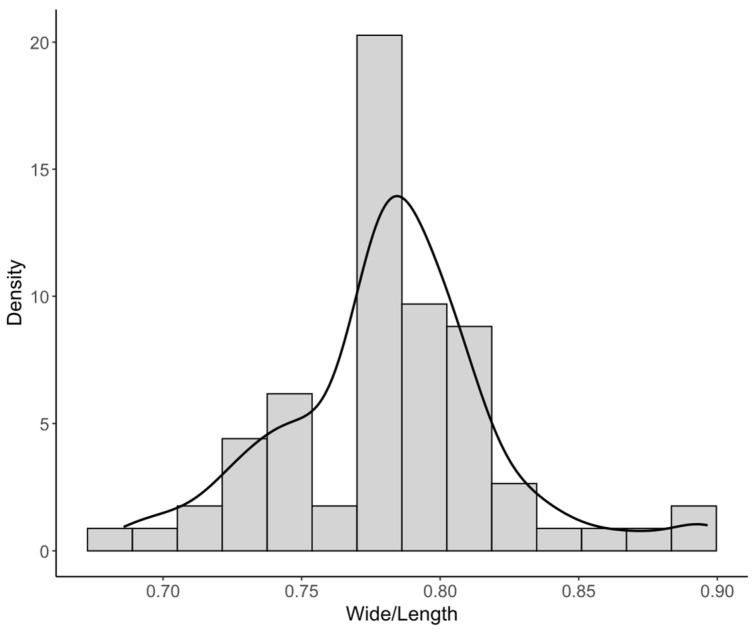
Distribution of the width-to-length ratio of CNC measured from TEM micrographs. The histograms and superimposed kernel density curve are expressed on a density scale.

This pattern was also reflected in the histograms and kernel density curves (Figure 2 and Figure 3). The distributions of length and width were positively skewed, with a predominance of small particles and a tail extending toward larger values. By contrast, the width-to-length ratio has a narrower unimodal distribution centered at 0.78, indicating a relatively consistent geometric proportion among the analyzed nanoparticles. Overall, these results show that the CNC population was polydisperse in absolute dimensions while maintaining a comparatively stable aspect ratio.

### 3.2. Phenolic Compounds

Phenolic fractions, including free phenols, condensed tannins, and hydrolyzed tannins, were quantified in chickpea flour and in thermocompressed chickpea sheets (Table 2). Thermocompression significantly reduced the content of all phenolic fractions compared with flour (*p* < 0.05). Free phenols decreased from 0.813 ± 0.116 to 0.556 ± 0.023 mg GAE g^−1^ sample, condensed tannins from 4.197 ± 0.609 to 2.259 ± 0.157 mg CAE g^−1^ sample, and hydrolyzed tannins from 1.121 ± 0.086 to 0.969 ± 0.022 mg GAE g^−1^ sample (flour vs. sheet, respectively). Among the analyzed fractions, condensed tannins were the predominant phenolic fraction in both materials, followed by hydrolyzed tannins, whereas free phenols were the lowest fraction (Table 2).

These results indicate that thermocompression reduced the measurable phenolic content of the chickpea matrix, although the relative distribution of phenol fractions remained unchanged.

### 3.3. Antioxidant Capacity (DPPH Radical Scavenging)

Antioxidant capacity, determined by the DPPH assay and expressed as RSA and % inhibition, is shown in Table 3 for the chickpea flour and thermocompressed chickpea sheet. No significant differences were observed between flour and sheets within any of the evaluated phenolic fractions (*p* > 0.05).

The condensed tannin fraction showed the highest RSA values in both materials, reaching 84.48 ± 2.72% in flour and 83.86 ± 1.04% in sheets. The hydrolyzable tannin fraction showed intermediate antioxidant activity, whereas the free phenolic fraction exhibited the lowest RSA values. These results indicate that thermocompression did not significantly affect the DPPH radical-scavenging capacity of the extracted phenolic fractions, despite the reductions observed in phenolic content.

The hydrolyzable tannin fraction of the sheets showed greater variability in RSA than the corresponding flour fraction, indicating a less consistent antioxidant response after thermocompression and extraction. Therefore, this result should be interpreted with caution.

### 3.4. Morphology of Thermocompressed Sheets (SEM)

**Figure 4:**
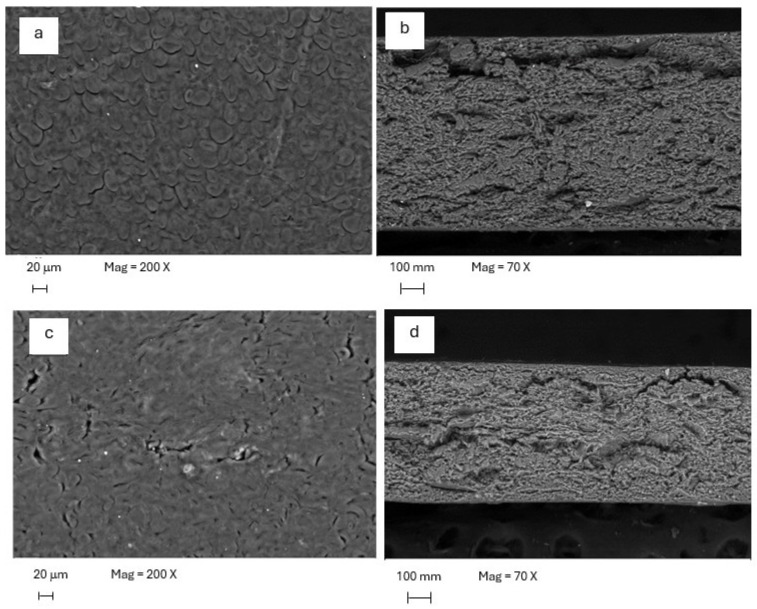
SEM micrographs of thermocompressed chickpea sheets: (**a**) control surface (200×; 20 µm scale bar); (**b**) control cross-section (70×; 100 µm scale bar); (**c**) 7.5% CNC-containing sheet surface (200×; 20 µm scale bar); (**d**) 7.5% CNC-containing sheet cross-section (70×; 100 µm scale bar).

SEM micrographs of thermocompressed chickpea sheets are shown in Figure 4. The surface of the control sheet exhibited a relatively uniform morphology, with granular features clearly distinguishable. Its cross-section shows a consolidated and continuous matrix with a compact appearance. In contrast, the sheet containing 7.5% CNC displayed a smoother surface topography, with less evidence of granular features. The corresponding cross-section showed a rougher fracture profile than that of the control sheet.

These observations indicate that incorporating 7.5% CNC modified the surface appearance and the fracture morphology of the thermocompressed sheets. However, due to the low magnification used, SEM was not considered suitable for direct assessment of nanoscale CNC distribution within the matrix. Therefore, the SEM results were interpreted only as evidence of changes in surface topography and fracture morphology between formulations.

### 3.5. Mechanical Properties

The mechanical properties of thermocompressed chickpea flour sheets reinforced with different CNC concentrations are shown in Table 4. The tensile strength (TS) increased with increasing CNC content, from 2.11 ± 0.56 MPa in the control sheet to 3.10 ± 0.36 MPa in the formulation containing 7.5% CNC. The control sample exhibited the lowest TS and differed significantly from the CNC-containing sheets (*p* < 0.05), whereas the 2.5% and 5.0% CNC treatments exhibited intermediate values.

The elongation at break $\left(\varepsilon_{b}\right)$ increased at 2.5% and 5.0% of CNC, reaching 8.28 ± 1.35% and 9.22 ± 0.74%, respectively, compared with 6.18 ± 1.84% for the control. At 7.5% CNC, $\varepsilon_{b}$ decreased to 6.68 ± 1.25%, returning to a value that did not differ significantly from the control. These results indicate that intermediate CNC concentrations favored greater sheet extensibility, whereas this effect was not maintained at the highest CNC load.

Regarding stiffness, Young’s modulus remained statistically unchanged at 2.5% and 5.0% CNC relative to the control but increased significantly at 7.5% CNC to 1.35 ± 0.16 MPa. Overall, these results indicate that CNC reinforcement improved the mechanical strength of the sheets, while the highest CNC concentration increased stiffness without preserving the extensibility gains observed at intermediate loadings.

### 3.6. Water Vapor Permeability (WVP)

WVP values of thermocompressed chickpea flour sheets decreased significantly with increasing CNC concentrations (Table 5). The control formulation showed the highest WVP, whereas the sheet containing 7.5% CNC exhibited the lowest WVP. Intermediate CNC concentrations (2.5% and 5.0%) produced a gradual reduction in WVP, indicating that nanocellulose improved the sheets’ barrier performance in a concentration-dependent manner.

The incorporation of 2.5% and 5.0% CNC resulted in intermediate WVP values, indicating a concentration-dependent reduction in WVP. Overall, these results show that CNC addition improved the WVP barrier properties of the chickpea sheets.

### 3.7. Optical Properties

#### 3.7.1. Color

CIELAB-derived color parameters (L*, Chroma, and Hue) of thermocompressed chickpea sheets as a function of CNC loading are presented in Table 6. All three parameters increased significantly with increasing CNC concentration (*p* < 0.05), indicating that CNC incorporation modified the optical appearance of the sheets.

Lightness (L*) increased from 40.00 ± 0.55 in the control to 55.22 ± 1.65 in the formulation containing 7.5% CNC, with intermediate values observed at 2.5% and 5.0% CNC. A similar trend was observed for Chroma, which increased from 23.62 ± 0.12 in the control to 44.86 ± 1.35 at 7.5% CNC, indicating a progressive intensification of color. Hue also increased with CNC concentration, from 41.81 ± 4.47 in the control to 61.10 ± 2.63 at 7.5% CNC. The 5.0% and 7.5% CNC formulations did not differ significantly in Hue, although both were significantly higher than the control and 2.5% CNC treatments.

Overall, these results show that increasing CNC concentration produced lighter sheets with higher Chroma and higher hue values, indicating a clear modification of sheet color as a function of nanocellulose loading.

#### 3.7.2. Transparency and Opacity

Opacity and transmittance at 600 nm (%T_600_) of thermocompressed chickpea sheets as a function of CNC loading are presented in Table 7. Opacity increased with increasing CNC concentration. The control (0% CNC) and 2.5% CNC sheets showed the lowest opacity values (2.21 ± 0.15 and 2.26 ± 0.04 AU·mm^−1^, respectively) and did not differ significantly from each other. In contrast, the formulations containing 5.0% and 7.5% CNC exhibited significantly higher opacity values (3.04 ± 0.08 and 3.15 ± 0.06 AU·mm^−1^, respectively; *p* < 0.05).

The %T_600_ values were low for all formulations, ranging from 2.3 ± 0.141% to 4.05 ± 0.806%, indicating limited visible-light transmission through the sheet. The 2.5% CNC formulation showed the lowest %T_600_ value and differed significantly from the 5.0% and 7.5% CNC (*p* < 0.05). In contrast, the control showed an intermediate value and did not differ significantly from the other treatments.

Overall, these results indicate that CNC incorporation modified the optical response of the thermocompressed chickpea sheets. In particular, higher CNC concentrations increased the thickness-normalized opacity, although the direct transmittance data showed a non-monotonic pattern, with the lowest mean light transmission at 2.5% CNC.

### 3.8. Thermal Properties

**Figure 5:**
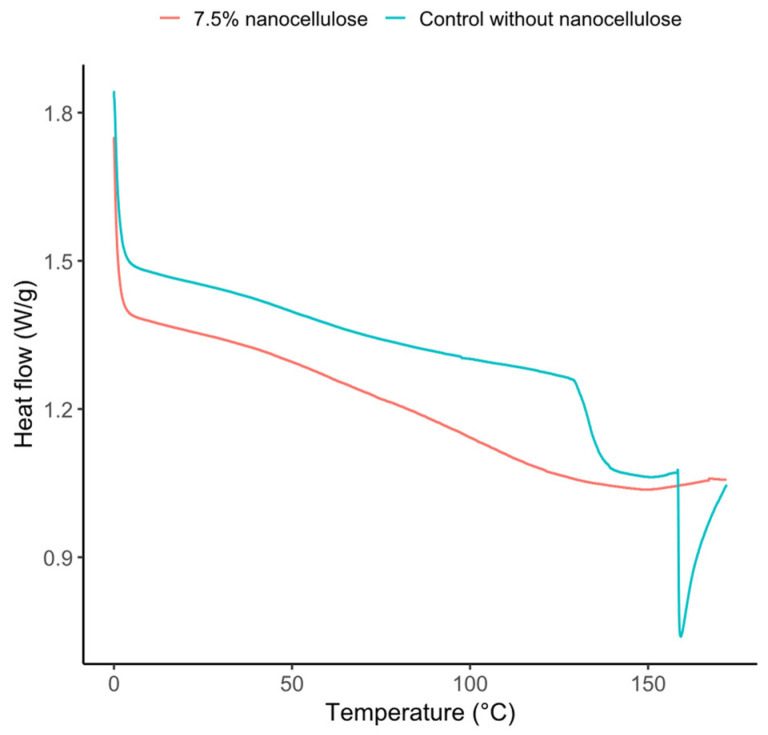
DSC thermograms of chickpea flour sheets, control, and 7.5% CNC.

The DSC thermograms of thermocompressed chickpea sheets are shown in Figure 5, and the corresponding thermal parameters are summarized in Table 8. The control sheet exhibited a sharp endothermic event, with an onset temperature (Tonset) of 127.4 °C, a peak temperature (Tpeak) of 133.7 °C, and an endset temperature (Tendset) of 143.0 °C. The enthalpy associated with this event was 64.0 J/g. In contrast, the sheet containing 7.5% CNC showed a broader, less-defined thermal event, with Tonset, Tpeak, and Tendset values of 92.0 °C, 147.9 °C, and 166.4 °C, respectively, and a markedly lower enthalpy (15.7 J/g). These results indicate that incorporating CNC modified the thermal response of the chickpea sheet matrix, broadening the main thermal event and reducing its intensity.

**Figure 6:**
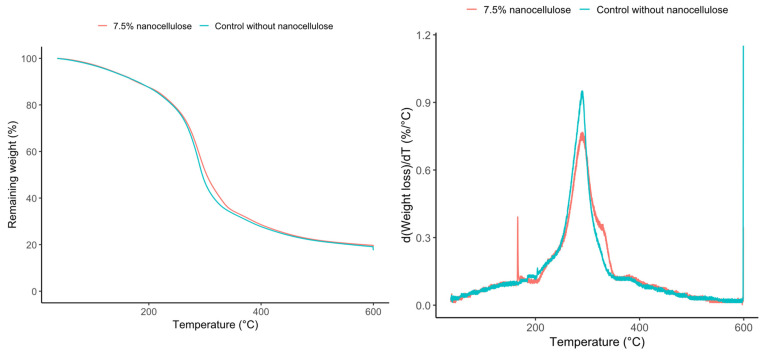
TGA and DTGA analysis of chickpea flour sheet, control, and 7.5% CNC.

The thermogravimetric behavior of the sheets is shown in Figure 6, and the main TGA parameters are presented in Table 9. Both the control and the sheet containing 7.5% nanocellulose exhibited similar thermal degradation behavior. The temperature at 5% weight loss (T5%) was 126 °C for both formulations, and the temperature at 10% weight loss (T10%) was 180 °C in both cases. Likewise, the onset degradation temperature (Tonset) increased only slightly from 215.0 °C in the control to 215.5 °C in the CNC-containing sheet. The maximum degradation temperature (Tmax) also increased slightly, from 287.0 °C in the control to 288.9 °C in the sheet with 7.5% CNC. The residual mass at 600 °C was the same for both samples (19.48%). Overall, these results indicate that CNC incorporation produced only minor changes in the thermal degradation profile of the sheets under the conditions evaluated. A more detailed examination of the DTGA profiles provides further insight into the degradation process. In the sheet containing 7.5% CNC, the shoulder observed at approximately 300–350 °C may be associated with the degradation of the cellulosic nanophase introduced by cellulose nanocrystals [36]. By contrast, the peak observed around 380 °C in both formulations may be associated with a later degradation stage of the flour-based matrix and/or with the more thermally stable residue generated during the decomposition of the starch-rich matrix [37,38]. Therefore, the shoulder in the reinforced sheet suggests an additional contribution from CNC to the degradation profile. In contrast, the peak near 380 °C appears to be a common feature of the thermocompressed chickpea-flour matrix [36,37,38].

T5%, temperature at 5% weight loss; T10%, temperature at 10% weight loss; Tmax, temperature of maximum degradation rate.

### 3.9. Structural Characterization

#### 3.9.1. XRD

**Figure 7:**
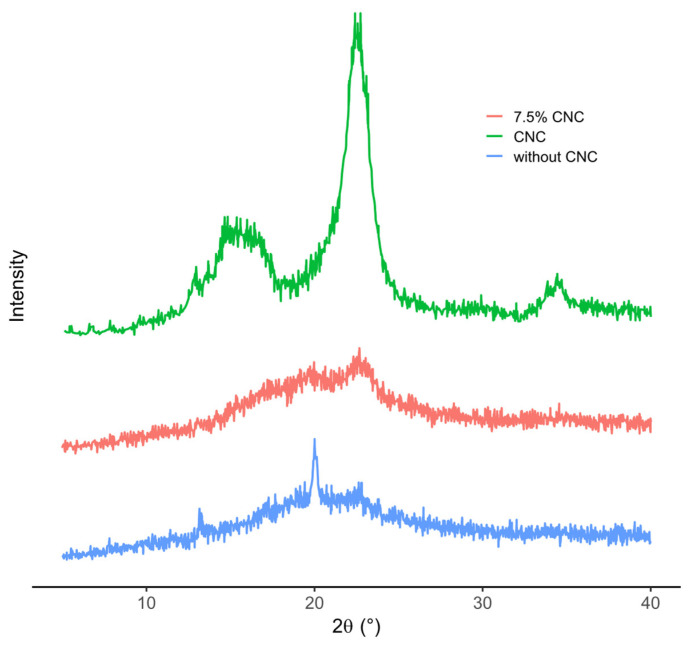
XRD patterns of CNC, thermocompressed chickpea sheet without CNC (control), and chickpea sheet containing 7.5% CNC.

XRD patterns of the control sheet (without CNC), the sheet containing 7.5% CNC, and the isolated CNC sample are shown in Figure 7. The CNC diffractogram exhibited a dominant reflection at approximately 2θ≈22–23°, together with a broader contribution in the ∼14–16° region and a minor feature around ∼34–35°. In contrast, the control sheets showed a broad diffraction halo centered in the ∼20–22°, indicating the predominance of an amorphous structure with a limited ordered contribution.

The sheet containing 7.5% CNC retained a broad diffraction band in the ∼20–23° region, but with higher relative intensity than the control. However, its pattern remained less defined than that of the isolated CNC sample. These results indicate that CNC incorporation modified the diffraction profile of the thermocompressed chickpea sheet, suggesting a structural contribution of the nanocellulose phase to the composite matrix.

#### 3.9.2. FTIR

**Figure 8:**
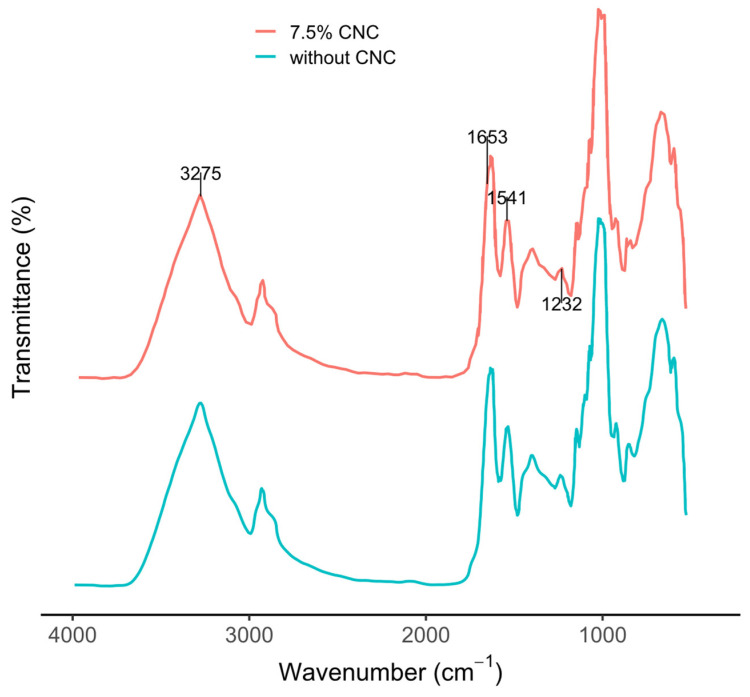
FTIR spectra of thermocompressed chickpea sheets without CNC (control) and with 7.5% CNC.

FTIR spectra of thermocompressed chickpea sheets with and without CNC are shown in Figure 8. Both spectra exhibited a broad band in the 3200–3400 cm^−1^ region, with a maximum near 3275 cm^−1^, assigned mainly to O-H stretching vibrations of hydroxyl groups from polysaccharide/cellulose components, overlapping with N-H stretching (amide A) from constituents of the chickpea matrix. According to the literature, for isolated cellulose nanocrystals prepared by sulfuric acid hydrolysis, the broad, high-intensity band in the 3200–3500 cm^−1^ region is consistently assigned to O-H stretching associated with intra- and intermolecular hydrogen bonding in cellulose [39,40,41].

In the sheet containing 7.5% CNC, the band at 1653 cm^−1^ was assigned to amide I (C=O stretching), whereas the band at 1541 cm^−1^ was assigned to amide II (N-H bending coupled with C-N stretching), both associated with protein-related structures present in the chickpea matrix. The band at 1232 cm^−1^ was assigned to vibrations associated with polysaccharide- and cellulose-related structures. Literature reports for CNC also describe C-O-C stretching near 1158–1162 cm^−1^, C-O stretching bands in the 1023–1120 cm^−1^ region, and β-(1 → 4)-glycosidic linkage vibrations near 897–898 cm^−1^ [36,39,40,42].

For sulfuric-acid-hydrolyzed CNC, additional sulfate ester-related bands have been reported in the 1010–1080 cm^−1^, 1150–1260 cm^−1^, and approximately 1205 cm^−1^ regions, and these bands are considered diagnostic markers of sulfuric acid treatment [39,42]. Therefore, the difference observed in the fingerprint region between the control and the 7.5% CNC sheets was interpreted as consistent with a greater relative contribution of cellulose-related vibrations after CNC incorporation.

An isolated FTIR spectrum was not acquired in the present study; comparisons with characteristic CNC bands were based on literature reports. Accordingly, the FTIR results were interpreted conservatively as indicating changes in the relative contributions and organization of hydroxyl, amide, and glycosidic functional groups, rather than as evidence of the formation of new chemical species. This interpretation is consistent with the literature, which states that CNC spectra retain the basic cellulose structure after acid treatment and do not necessarily indicate new bond formation [39,40].

## 4. Discussion

The present results are consistent with the working hypothesis that CNC can improve the barrier and thermo-mechanical performance of thermocompressed chickpea-flour sheets by promoting a more tortuous diffusion pathway and a more organized matrix. The selection of 22% moisture as the reference processing condition was justified by the improved handling and lamination observed during thermocompression, which enabled the preparation of continuous sheets under reproducible conditions. Under this processing route, CNC incorporation produced systematic changes in barrier, mechanical, optical, thermal, and structural behavior, suggesting that the nanofiller contributed to the reorganization of the chickpea-flour matrix during consolidation under heat and pressure. This interpretation is consistent with the expected role of CNC as a rigid, hydroxyl-rich nanofiller with high crystallinity and high specific surface area, capable of strengthening hydrophilic polymer networks and increasing diffusion tortuosity in bio-based composites [10,12,15,16,17,18].

The CNCs obtained in this study were clearly in the nanometric range, although they exhibited broad dispersion in absolute dimensions and much lower variability in the width-to-length ratio. This combination suggests that the nanocrystals were polydisperse in size but relatively stable in geometric proportion, which is relevant because reinforcement efficiency depends not only on particle size, but also on aspect ratio, dispersion state, and interfacial development within the matrix [15,16,17,18]. In chickpea-flour systems, where proteins, carbohydrates, and minor lipids coexist, such nanometric particles are expected to promote physical interactions and local structural rearrangements during thermocompression, thereby affecting both mechanical resistance and mass transfer.

The mechanical results are consistent with the reinforcing role of CNC in hydrophilic polymer matrices. Cellulose nanocrystals are highly crystalline, rigid nanoparticles with a large specific surface area and hydroxyl-rich surfaces, which favor physical interactions with the chickpea-flour matrix, particularly with its polysaccharide and protein components. In this type of system, CNC can facilitate stress transfer within the composite, reduce segmental mobility, and promote a denser, more cohesive network. These effects help explain the progressive increase in tensile strength with CNC loading and the significant increase in Young’s modulus at 7.5% CNC. At the same time, the reduction in elongation at break at the highest CNC level suggests that the reinforced network became less deformable, which is consistent with the restricted chain mobility commonly reported for CNC-containing composites. Similar reinforcing behavior has been described in polymer systems containing cellulose nanocrystals, where the balance between improved strength and reduced deformability depends on filler content, dispersion state, and matrix–filler interactions [15,16,17,43]. From an application standpoint, the combination of increased stiffness, improved tensile strength, and reduced water-vapor permeability suggests that the developed sheets may be better suited to semirigid bio-based packaging uses, such as trays, inserts, dividers, or secondary packaging for low-moisture foods, than to highly flexible packaging formats.

The most pronounced functional enhancement was observed in WVP. It decreased from 5.16 × 10^−10^ g·m^−1^·s^−1^·Pa^−1^ in the control to 1.41 ×10^−10^, 6.79 × 10^−11^, and 5.93 × 10^−12^ g·m^−1^·s^−1^·Pa^−1^ at 2.5, 5.0, and 7.5% CNC, respectively, with significant differences among all treatments. This strong, concentration-dependent reduction indicates that CNC modified the water-vapor transport pathway within the sheet matrix. From a mechanistic perspective, the result is consistent with a combined effect of increased tortuosity, reduced free volume, and matrix densification induced by the nanofiller [13,15,16]. A similar barrier-improving effect of CNC has also been reported in other legume- and protein-based matrices, including faba bean protein films. In contrast, CNC reduced water-vapor transmission and improved overall barrier performance [44]. Therefore, the present results support the view that CNC reinforcement can effectively enhance the moisture barrier of thermocompressed chickpea-flour sheets. However, the magnitude of the response is also likely influenced by the processing route.

The SEM observations provide morphological support for differences between the control and the 7.5% CNC sheet. The control showed more evidence of granular features, whereas the CNC-containing sheet exhibited a smoother surface and a modified fracture profile. However, because the images were obtained at low magnification, SEM does not permit direct visualization of nanoscale CNC dispersion or direct confirmation of nanoscale matrix reorganization. Therefore, these observations were interpreted only as evidence of changes in surface topography and fracture morphology, while the broader structural interpretation relied on the combined evidence from XRD, FTIR, and thermal analysis.

A smoother surface and a more integrated fracture morphology are compatible with a denser structure, which would contribute to the lower WVP, and higher stiffness observed at higher CNC loadings. Thus, the morphological evidence supports the interpretation that CNC acted as a structural modifier of the matrix rather than merely as an inert particulate additive.

The optical results also reflected changes in sheet appearance and light attenuation. L*, Chroma, and Hue all increased with CNC concentration, indicating that CNC incorporation modified the visual appearance of the sheets. At the same time, all formulations exhibited low transmittance at 600 nm (%T_600_ =2.3–4.05%), indicating limited visible-light transmission through the material. The lowest mean %T_600_ value was observed at 2.5% CNC, whereas the 5.0% and 7.5% CNC sheets showed the highest values. However, the control sheet displayed an intermediate transmittance and did not differ significantly from the other treatments.

In contrast, opacity increased significantly at 5.0% and 7.5% CNC, indicating greater light attenuation on a thickness-normalized basis at higher nanocellulose loadings. These results suggest that CNC altered the optical response of the sheets, although neither direct light transmittance nor thickness-normalized opacity followed the same trend. From an application standpoint, the low visible-light transmittance and increased opacity at higher CNC loadings may be advantageous for light-sensitive products. However, they may reduce the material’s visual clarity.

The thermal analyses, performed for the control and the 7.5% CNC sheet, revealed a more nuanced effect of CNC. In DSC, the control sheet exhibited a sharp endothermic event (Tonset = 127.4 °C, Tpeak = 133.7 °C, Tendset = 143.0 °C) with an enthalpy of 64.0 J/g. In contrast, the sheet containing 7.5% CNC showed a broader and less defined event (Tonset = 92.0 °C, Tpeak = 147.9 °C, Tendset = 166.4 °C) with a much lower enthalpy (15.7 J/g). This result indicates that, at the highest CNC loading evaluated by DSC, the nanofiller modified the thermal transition profile of the chickpea matrix, broadening the main event and reducing its intensity. Such behavior is compatible with a reorganization and altered chain mobility in the thermocompressed network [16,17]. In contrast, TGA showed only minor differences between the control and the 7.5% CNC formulation: T5% and T10% were unchanged, Tonset and Tmax increased only slightly, and the residual mass at 600 °C was identical. Therefore, the data support a stronger claim for modification of the DSC thermal response than for a substantial increase in degradation resistance.

The structural analyses by XRD and FTIR, also performed for the control and 7.5% CNC sheet, reinforce this interpretation. The isolated CNC sample exhibited a dominant reflection at approximately 2θ ≈ 22–23°, together with a broader contribution in the 14–16° region and a minor feature around 34–35°, whereas the control sheet showed a broad halo centered in the 20–22° region, consistent with a predominantly amorphous matrix. The sheet containing 7.5% CNC retained a broad band in the 20–23° region, but with higher relative intensity than the control, indicating that the nanocellulose phase contributed to the structural organization of the composite without fully transforming it into a highly ordered material. Likewise, FTIR spectra support compositional and structural differences between formulations. The broad band near 3275 cm^−1^ was associated with overlapping hydroxyl and amide A vibrations. In contrast, the bands at 1653 and 1541 cm^−1^ corresponded to amide I and amide II contributions from protein-related groups in the chickpea matrix. The fingerprint-region changes, including the band at 1232 cm^−1^, were interpreted in light of literature-reported CNC bands assigned to glycosidic C-O-C stretching, C-O stretching, and β-(1→4)-glycosidic linkage vibrations, as well as sulfate ester-related bands characteristic of sulfuric-acid-hydrolyzed CNC [36,39,40,42]. Because an isolated CNC FTIR spectrum was not available in the present study, these assignments were compared with literature values, and the interpretation was limited to changes in the relative contributions and organization of pre-existing functional groups rather than the formation of new chemical species.

The phenolic and antioxidant results provide additional insight into the effect of thermocompression on the chickpea matrix. Thermocompression reduced the measurable content of free phenols, condensed tannins, and hydrolyzable tannins. However, the DPPH radical-scavenging activity of the extracted fractions did not change significantly between flour and sheets. This suggests that thermocompression may have affected the extractability or matrix association of phenolic compounds more strongly than their measurable antioxidant functionality. The higher variability observed for the hydrolyzable tannin fraction in sheet samples also points to matrix-related heterogeneity affecting extraction and antioxidant measurements after thermocompression. It should also be noted that condensed tannins were estimated using the butanol-HCl-Fe assay and expressed as catechin equivalents; therefore, these values should be interpreted as assay-based estimates of this phenolic fraction. Previous studies have shown that this method may underestimate bound condensed tannins, so the results should be interpreted with caution [25]. Overall, these findings indicate that the processing route did not produce a proportional loss of antioxidant response, which is favorable from a functional standpoint. In practical terms, the processing route used to form the sheets did not translate into a proportional loss of antioxidant response, which is favorable from a functional standpoint.

Generally, the present results indicate that CNC reinforcement in thermocompressed chickpea-flour sheets is associated with matrix densification, structural reorganization, and increased diffusion tortuosity. This mechanism explains why the most evident improvements were observed in WVP, tensile strength, and Young’s modulus, whereas the effect on TGA stability was comparatively limited. From a practical perspective, these sheets appear more suitable for semirigid bio-based packaging applications, particularly in systems where reduced water-vapor transport and moderate structural rigidity are desirable, rather than in highly flexible packaging formats. Because the formulations are based on chickpea flour, glycerol, and CNCs, they are compositionally compatible with biodegradable-material approaches; however, their compostability and suitability for organic recycling were not evaluated in this study and should be addressed in future work. Although the formulations are based on bio-derived components, the present study did not evaluate migration, toxicological safety, or regulatory compliance; therefore, any direct food-contact or edible application would require additional assessment under the relevant food-contact regulatory frameworks before practical implementation. These findings are relevant because most previous studies on chickpea-based matrices reinforced with CNC have focused on cast films. At the same time, the present work demonstrates that thermocompression can also generate a meaningful reinforcement response in legume-flour sheets [10,11,19,20]. In practical terms, CNC is an effective reinforcing phase when the design objective is to reduce water vapor transport while maintaining or improving tensile performance under thermocompression processing.

Future studies should quantify the degree of structural order from XRD, strengthen FTIR band assignments, and evaluate additional performance indicators such as oxygen permeability, long-term storage stability, and high-resolution filler dispersion. It would also be valuable to clarify whether the observed reduction in phenolic content is due to degradation, stronger matrix binding, or reduced extraction efficiency. Taken together, the current results support CNC as an effective reinforcing phase for thermocompressed chickpea-flour sheets, especially when the main objective is to improve water-vapor barrier performance while increasing mechanical strength and modifying the thermo-structural response of the matrix.

Taken together, the current results support CNC as an effective reinforcement phase for thermocompressed chickpea-flour sheets, particularly when the main objective is to improve water-vapor barrier performance while increasing mechanical strength and modifying the thermo-structural response of the matrix.

## 5. Conclusions

Cellulose nanocrystals (CNCs) were successfully incorporated into thermocompressed chickpea flour sheets, significantly modifying their physicochemical, mechanical, barrier, optical, thermal, and structural properties. The most relevant effect of CNC addition was the strong reduction in water vapor permeability, which decreased progressively with increasing CNC concentration, indicating a clear improvement in barrier performance. Mechanical properties were also enhanced: tensile strength increased with CNC loading, and Young’s modulus was significantly higher at 7.5% CNC. However, elongation at break reached its highest values at intermediate CNC concentrations and decreased at the highest loading.

Optical characterization showed that CNC incorporation modified visible-light transmittance and opacity. Thermal analysis showed that CNC altered the main DSC thermal event, broadening the transition and reducing its enthalpy, whereas only minor differences were observed in the TGA degradation profile. Structural analyses by SEM, XRD, and FTIR suggested changes in morphology and thermo-structural organization after CNC incorporation. In addition, although thermocompression reduced the measurable phenolic fractions, the antioxidant activity of the extracted fractions was not significantly affected.

Overall, these results show that CNC is an effective reinforcing phase for thermocompressed chickpea flour sheets, particularly when the objective is to improve water-vapor barrier performance and tensile behavior in a bio-based material intended for semirigid packaging applications, such as trays, inserts, dividers, or secondary packaging for low-moisture foods. However, further studies are required to evaluate migration behavior, toxicological safety, regulatory suitability, and end-of-life performance before any direct food-contact or edible application is proposed.

## Figures and Tables

**Table 1:** Descriptive summary of CNC dimensions measured from TEM micrographs. Because length and width distributions were right-skewed, the median (Q1–Q3) is reported as the primary descriptor; mean ± SD is included for comparison.

	n	Median (Q1–Q3) (nm)	Min–Max (nm)	Mean ± SD (nm)	CV (%)
Length	70	8.94 (6.20–29.94)	3.82–66.83	17.23 ±15.72	91.24
Width	70	6.82 (4.92–23.53)	2.86–58.21	13.58 ± 12.72	93.62
Width/Length ratio	70	0.784 (0.763–0.803)	0.686–0.897	0.782 ± 0.040	5.07

n, number of nanoparticles analyzed; SD, standard deviation; Q1, first quartile; Q3, third quartile; CV, coefficient of variation.

**Table 2:** Phenolic fractions in chickpea flour and thermocompressed chickpea sheets (mean ± SD, n = 3).

	Flour	Sheets
Free Phenol (mg GAE/g sample)	0.813 ± 0.116 ^a^	0.556 ± 0.023 ^b^
Condensed Tannins (mg CAE/g sample)	4.197 ± 0.609 ^c^	2.259 ± 0.157 ^d^
Hydrolyzed Tannins (mg GAE/g sample)	1.121 ± 0.086 ^e^	0.969 ± 0.022 ^f^

Different letters within the same row indicate significant differences between flour and sheets (*p* < 0.05).

**Table 3:** Radical-scavenging activity of phenolic fractions extracted from chickpea flour and thermocompressed chickpea sheets (mean ± SD, n = 3).

	Flour (RSA % Inhibition)	Sheets (RSA % Inhibition)
Free Phenol	22.12 ± 1.85 ^a^	20.80 ± 0.19 ^a^
Condensed Tannins	84.48 ± 2.72 ^b^	83.86 ± 1.04 ^b^
Hydrolyzed Tannins	76.87 ± 2.43 ^c^	66.31 ± 12.88 ^c^

Different letters within the same row indicate significant differences between flour and sheets (*p* < 0.05).

**Table 4:** Mechanical properties of thermocompressed chickpea flour sheets reinforced with different CNC contents (mean ± SD; n = 10).

CNC (%)	Tensile Strength (MPa)	$\varepsilon_{b}$ (%)	Young’s Modulus (MPa)
0	2.11 ± 0.56 ^a^	6.18 ± 1.84 ^a^	0.88 ± 0.07 ^a^
2.5	2.67 ± 0.28 ^b^	8.28 ± 1.35 ^b^	0.97 ± 0.14 ^a^
5.0	2.80 ± 0.27 ^b,c^	9.22 ± 0.74 ^b^	0.94 ± 0.11 ^a^
7.5	3.10 ± 0.36 ^c^	6.68 ± 1.25 ^a^	1.35 ± 0.16 ^b^

Different lowercase letters within the same column indicate significant differences among treatments (*p* < 0.05).

**Table 5:** Water vapor permeability of thermocompressed chickpea flour sheets reinforced with different CNC concentrations (mean ± SD; n = 3).

CNC Concentration (%)	WVP $\left(g\cdot m^{-1}s^{-1}Pa^{-1}\right)$
0.0	$5.16\times 10^{-10}\pm 3.65\times 10^{-11}$ ^a^
2.5	$1.41\times 10^{-10}\pm 6.16\times 10^{-12}$ ^b^
5.0	$6.79\times 10^{-11}\pm 6.70\times 10^{-12}$ ^c^
7.5	$5.93\times 10^{-12}\pm 2.77\times 10^{-13}$ ^d^

Different letters within the same column indicate a significant difference among treatments (*p* < 0.05).

**Table 6:** CIELAB color parameters of thermocompressed chickpea flour sheets reinforced with different CNC concentrations (mean ± SD; n = 4).

CNC (%)	L*	Chroma	Hue
0	40.00 ± 0.55 ^a^	23.62 ± 0.12 ^a^	41.81 ± 4.47 ^a^
2.5	41.16 ± 0.94 ^a^	25.29 ± 1.36 ^a^	48.77 ± 1.59 ^a^
5.0	52.40 ± 0.72 ^b^	41.41 ± 1.00 ^b^	59.27 ± 1.08 ^b^
7.5	55.22 ± 1.65 ^c^	44.86 ± 1.35 ^c^	61.10 ± 2.63 ^b^

Different lowercase letters within the same column indicate significant differences among treatments (ANOVA/Tukey, α = 0.05).

**Table 7:** Opacity and transmittance at 600 nm of thermocompressed chickpea flour sheets reinforced with different CNC concentrations (mean ± SD; n = 4).

CNC (%)	Opacity (AU. mm^−1^)	Transmittance at 600 nm (%T_600_)
0	2.21 ± 0.15 ^a^	3.40 ± 0.337 ^ab^
2.5	2.26 ± 0.04 ^a^	2.30 ± 0.141 ^b^
5.0	3.04 ± 0.08 ^b^	3.94 ± 0.677 ^a^
7.5	3.15 ± 0.06 ^b^	4.05 ± 0.806 ^a^

Different lowercase letters within the same column indicate significant differences among treatments (ANOVA/Tukey, α = 0.05).

**Table 8:** DSC thermal parameters of thermocompressed chickpea sheets with and without 7.5% CNC.

Sample	Event Description	T_onset_ (°C)	T_peak_ (°C)	T_end_set_ (°C)	Enthalpy (J/g)
Control without CNC	Sharp endothermic	127.4	133.7	143.0	64.0
7.5% CNC	Broad and less defined thermal event	92.0	147.9	166.4	15.7

T_onset_, onset temperature; T_peak_, peak temperature; T_endset_, endset temperature.

**Table 9:** TGA thermal parameters of thermocompressed chickpea sheets with and without 7.5% CNC.

Sample	T5% (°C)	T10% (°C)	Tonset (°C)	Tmax (°C)	Residual 600 °C (%)
Control without nanocellulose	126	180	215.0	287.0	19.48
7.5% nanocellulose	126	180	215.5	288.9	19.48

## Data Availability

The data presented in this study are openly available in figshare at https://doi.org/10.6084/m9.figshare.31920246.

## Abbreviations

The following abbreviations are used in this manuscript:

ASTM	American Society for Testing and Materials
ATR	Attenuated Total Reflectance
CIELAB	Commission International de l’Éclairage L*a*b* color space
CNC	Cellulose Nanocrystal
CS	Control Sheet
CSN	CNC-reinforced Sheet
CV	Coefficient of Variation
DPPH	2,2-Diphenyl-1-picrylhydrazyl
DSC	Differential Scanning Calorimetry
DTG	Derivative thermogravimetry
$\varepsilon_{b}$	Elongation at break
FTIR	Fourier Transform Infrared Spectroscopy
GAE	Gallic Acid Equivalents
HSD	Honestly Significant Difference
MWCO	Molecular Weight Cut-off
Q1	First Quartile
Q3	Third Quartile
RH	Relative Humidity
RSA	Radical-Scavenging Activity
SD	Standard Deviation
T5%	Temperature at 5% Weight Loss
T10%	Temperature at 10% Weight Loss
Tmax	Temperature of Maximum Degradation Rate
Tonset	Onset Temperature
Tpeak	Peak temperature
TS	Tensile Strength
WVP	Water Vapor Permeability
XRD	X-ray Diffraction

## References

[1] Canpolat M. (2024). Package-Borne Safety Issues in Food. Food Safety.

[2] Priyanka P., Rani R., Yadav R., Bajal N., Mandal B.S., Kumar A. (2024). Role of Active Packaging for Food Freshness and Quality Maintenance. Int. J. Environ. Agric. Biotechnol..

[3] Righetti G.I.C., Faedi F., Famulari A. (2024). Embracing Sustainability: The World of Bio-Based Polymers in a Mini Review. Polymers.

[4] Schäfer L. (2025). Is There Hope to Switch Traditional Plastics into Sustainable?. Sci. Insights.

[5] Long J., Zhang W., Zhao M., Ruan C.-Q. (2023). The Reduce of Water Vapor Permeability of Polysaccharide-Based Films in Food Packaging: A Comprehensive Review. Carbohydr. Polym..

[6] Czajkowska–González Y.A., Alvarez–Parrilla E., del Rocío Martínez–Ruiz N., Vázquez–Flores A.A., Gaytán–Martínez M., de la Rosa L.A. (2021). Addition of Phenolic Compounds to Bread: Antioxidant Benefits and Impact on Food Structure and Sensory Characteristics. Food Prod. Process. Nutr..

[7] Bigne F., Romero A., Ferrero C., Puppo M.C., Guerrero A. (2021). New Thermal and Rheological Approaches of Chickpea–Wheat Dough for Breadmaking. Eur. Food Res. Technol..

[8] Soto-Madrid D., Pérez N., Gutiérrez-Cutiño M., Matiacevich S., Zúñiga R.N. (2023). Structural and Physicochemical Characterization of Extracted Proteins Fractions from Chickpea (*Cicer arietinum* L.) as a Potential Food Ingredient to Replace Ovalbumin in Foams and Emulsions. Polymers.

[9] Jeya Jeevahan J., Chandrasekaran M., Venkatesan S.P., Sriram V., Britto Joseph G., Mageshwaran G., Durairaj R.B. (2020). Scaling up Difficulties and Commercial Aspects of Edible Films for Food Packaging: A Review. Trends Food Sci. Technol..

[10] Leya B., Franklin R.S., Pragalyaashree M.M., Monicka A.A., Tiroutchelvame D., Blessy C., Blessie R.F. (2024). Biopolymer-Based Edible Packaging: A Critical Review on the Biomaterials, Formation, and Applications on Food Products. J. Appl. Biol. Biotechnol..

[11] De Micheli C., Navarini F., Roncoroni V. (1995). Process for the Manufacture of Totally Bio-Decomposable Films with High Mechanical Characteristics and Relevant Products and Applications.

[12] Negrete-Bolagay D., Guerrero V.H. (2024). Opportunities and Challenges in the Application of Bioplastics: Perspectives from Formulation, Processing, and Performance. Polymers.

[13] Saravacos G.D., Karathanos V.T., Marousis S.N. (1992). Diffusion of Water in Starch Materials. Developments in Food Science.

[14] Alibekov R.S., Urazbayeva K.U., Azimov A.M., Rozman A.S., Hashim N., Maringgal B. (2024). Advances in Biodegradable Food Packaging Using Wheat-Based Materials: Fabrications and Innovations, Applications, Potentials, and Challenges. Foods.

[15] Sapkota J., Jorfi M., Weder C., Foster E.J. (2014). Reinforcing Poly(Ethylene) with Cellulose Nanocrystals. Macromol. Rapid Commun..

[16] Hung Y.-J., Chiang M.-Y., Wang E.-T., Wu T.-M. (2022). Synthesis, Characterization, and Physical Properties of Maleic Acid-Grafted Poly(Butylene Adipate-Co-Terephthalate)/Cellulose Nanocrystal Composites. Polymers.

[17] Duraccio D., Arrigo R., Strongone V., Capra P.P., Malucelli G. (2021). Rheological, Mechanical, Thermal and Electrical Properties of UHMWPE/CNC Composites. Cellulose.

[18] Liu G.-T., Shi S.-C., Rahmadiawan D. (2024). Revolutionizing Polymethyl Methacrylate Toughness: Achieving 190% Improvement with Nanocellulose Reinforcement While Maintaining Optical Clarity. J. Pendidik. Teknol. Kejuru..

[19] Le Duigou A., Deux J.-M., Davies P., Baley C. (2011). PLLA/Flax Mat/Balsa Bio-Sandwich Manufacture and Mechanical Properties. Appl. Compos. Mater..

[20] Åkesson D., Skrifvars M., Hagström B., Walkenström P., Seppälä J. (2009). Processing of Structural Composites from Biobased Thermoset Resins and Natural Fibres by Compression Moulding. J. Biobased Mater. Bioenergy.

[21] Lapuz A., Tsuchikawa S., Inagaki T., Ma T., Migo V. (2022). Production of Nanocellulose Film from Abaca Fibers. Crystals.

[22] R Core Team (2025). R: A Language and Enviromental for Statistical Computing.

[23] Wickham H. (2016). Ggplot2: Elegant Graphics for Data Analysis.

[24] Pérez-Jiménez J., Saura-Calixto F. (2005). Literature Data May Underestimate the Actual Antioxidant Capacity of Cereals. J. Agric. Food Chem..

[25] Makkar H.P.S., Gamble G., Becker K. (1999). Limitation of the Butanol–Hydrochloric Acid–Iron Assay for Bound Condensed Tannins. Food Chem..

[26] Hartzfeld P.W., Forkner R., Hunter M.D., Hagerman A.E. (2002). Determination of Hydrolyzable Tannins (Gallotannins and Ellagitannins) after Reaction with Potassium Iodate. J. Agric. Food Chem..

[27] Singleton V.L., Orthofer R., Lamuela-Raventós R.M., Packer L. (1999). Analysis of Total Phenols and Other Oxidation Substrates and Antioxidants by Means of Folin-Ciocalteu Reagent. Methods In Enzymology.

[28] Singleton V.L., Rossi J.A. (1965). Colorimetry of Total Phenolics with Phosphomolybdic-Phosphotungstic Acid Reagents. Am. J. Enol. Vitic..

[29] Brand-Williams W., Cuvelier M.E., Berset C. (1995). Use of a Free Radical Method to Evaluate Antioxidant Activity. LWT-Food Sci. Technol..

[30] Molyneux P. (2004). The Use of the Stable Free Radical Diphenylpicrylhydrazyl (Dpph) for Estimating Antioxidant Activity. Songklanakarin J. Sci. Technol. (SJST).

[31] Byun Y., Kim Y.T., Whiteside S. (2010). Characterization of an Antioxidant Polylactic Acid (PLA) Film Prepared with α-Tocopherol, BHT and Polyethylene Glycol Using Film Cast Extruder. J. Food Eng..

[32] (2002). Standard Test Method for Tensile Properties of Thin Plastic Sheeting.

[33] (2022). Standard Test Methods for Gravimetric Determination of Water Vapor Transmission Rate of Materials.

[34] Aguirre A., Mendez X., Borneo R. (2021). Characterization and Storage Study of Chickpea Flour Films with UV-Barrier and Cu-Remove Properties. Appl. Food Res..

[35] Zhao J., Wang Y., Liu C. (2022). Film Transparency and Opacity Measurements. Food Anal. Methods.

[36] Rana M.S., Rahim M.A., Mosharraf M.P., Tipu M.F.K., Chowdhury J.A., Haque M.R., Kabir S., Amran M.S., Chowdhury A.A. (2023). Morphological, Spectroscopic and Thermal Analysis of Cellulose Nanocrystals Extracted from Waste Jute Fiber by Acid Hydrolysis. Polymers.

[37] Kaczmarska K., Grabowska B., Grabowski G., Bobrowski A., Kurleto-Kozioł Ż. (2017). Thermal Decomposition of Binder Based on Etherified Starch to Use in Foundry Industry: TG–DTG–DSC and DRIFT Investigations. J. Therm. Anal. Calorim..

[38] Wang J., Sun X., Xu X., Sun Q., Li M., Wang Y., Xie F. (2022). Wheat Flour-Based Edible Films: Effect of Gluten on the Rheological Properties, Structure, and Film Characteristics. Int. J. Mol. Sci..

[39] Liu D., Liu J., Lin S., Wei X., Guo M. (2019). Preparation and Characterization of Cellulose Nanocrystals with Different Aspect Ratios as Nano-Composite Membrane for Cationic Dye Removal. SN Appl. Sci..

[40] Schiavi D., Ronchetti R., Di Lorenzo V., Vivani R., Giovagnoli S., Camaioni E., Balestra G.M. (2023). Sustainable Protocols for Cellulose Nanocrystals Synthesis from Tomato Waste and Their Antimicrobial Properties against *Pseudomonas syringae* Pv. Tomato. Plants.

[41] Surov O.V., Afineevskii A.V., Voronova M.I. (2023). Sulfuric Acid Alcoholysis as a Way to Obtain Cellulose Nanocrystals. Cellulose.

[42] Hussein Y., Kamoun E.A., Loutfy S.A., Amen R., Taha T.H., Mansour A.S., Abdel-Salam A.I., Amer M. (2020). Plant Nanocellulose and Its Composite Hydrogel Membranes-Based Polyvinyl Alcohol/Hyaluronic Acid for Biomedical Applications: Extraction, Characterization, and in Vitro Bioevaluation. J. Appl. Pharm. Sci..

[43] Maftoonazad N., Badii F., Mohamed A., Ramaswamy H. (2020). Evaluation of Physicochemical, Thermomechanical, and Structural Properties of Chickpea Flour Composite Films Reinforced with Crystalline Nanocellulose. J. Appl. Polym. Sci..

[44] Rojas-Lema S., Nilsson K., Trifol J., Langton M., Gomez-Caturla J., Balart R., Garcia-Garcia D., Moriana R. (2021). Faba Bean Protein Films Reinforced with Cellulose Nanocrystals as Edible Food Packaging Material. Food Hydrocoll..

